# Latexin Is Down-Regulated in Hematopoietic Malignancies and Restoration of Expression Inhibits Lymphoma Growth

**DOI:** 10.1371/journal.pone.0044979

**Published:** 2012-09-27

**Authors:** Yi Liu, Dianna Howard, Kyle Rector, Carol Swiderski, Jason Brandon, Lawrence Schook, Jayesh Mehta, J. Scott Bryson, Subbarao Bondada, Ying Liang

**Affiliations:** 1 Department of Internal Medicine, University of Kentucky College of Medicine, Lexington, Kentucky, United States of America; 2 Department of Physiology, University of Kentucky College of Medicine, Lexington, Kentucky, United States of America; 3 Department of Microbiology, Immunology and Molecular Genetics, University of Kentucky College of Medicine, Lexington, Kentucky, United States of America; 4 Department of Animal Sciences, University of Illinois, Urbana, Illinois, United States of America; 5 Department of Medicine, The Feinberg School of Medicine, Northwestern University, Chicago, Illinois, United States of America; University of Chicago, United States of America

## Abstract

Latexin is a negative regulator of hematopoietic stem cell number in mice. Its dysregulated expression in other tumors led us to hypothesize that latexin may have tumor suppressor properties in hematological malignancies. We found that latexin was down-regulated in a variety of leukemia and lymphoma cell lines as well as in CD34+ cells from the blood and marrow of patients with these malignancies. 5-aza-2′-deoxycytodine treatment and bisulfite sequencing revealed hypermethylation of latexin promoter in tumor cells. Retrovirus-mediated latexin overexpression in A20 mouse lymphoma cells inhibited their in vitro growth by 16 fold and in vivo tumor volume by 2 fold. Latexin caused growth inhibition of lymphoma cells by significantly increasing apoptosis through the down-regulation of anti-apoptotic genes Bcl-2 and Pim-2. The molecular mechanism underlying latexin-mediated tumor inhibition was not through its canonical carboxypeptidase inhibitor activity. These results are consistent with a tumor suppressor role for latexin and suggest that latexin may have clinical efficacy in the treatment of malignancies.

## Introduction

Both solid and liquid tumors are now understood to originate from the malignant transformation of resident adult stem and progenitor cells. [1], [2] Nowhere is this paradigm better established than in leukemia, yet events causing neoplastic conversion remain poorly understood. [3] We identified latexin (*Lxn*) as a novel, homeostatic regulator of the size of the hematopoietic stem cell population in mice. [4] The stem cell pool size was inversely related to quantitative *Lxn* expression at both the levels of transcript and protein. We found that population size was influenced by *Lxn* in a stem cell-autonomous manner, and acting through the concerted mechanisms of self-renewal and apoptosis, which were decreased and increased, respectively, by *Lxn* abundance. [4] Thus, *Lxn* acts as a brake on the expansion of stem cell population. Unrestrained stem cell expansion carries with it the risk of mutations, genomic instability and carcinogenesis. We therefore hypothesized that *Lxn* expression patterns in stem and progenitor cells may act as a tumor suppressor by inhibiting stem and progenitor cell proliferation, influencing the crucial steps toward malignancy.

*Lxn* was primarily studied in the nervous system, involving the specification of cortical brain regions during development [5], [6], [7], [8], [9], as well as the speed of nerve transmission in adult peripheral nervous systems [10]. *Lxn* was found to be involved in the inflammatory response in macrophages owing to its paired cystatin-like domains [11]. It was detected in mast cells associated with a unique type of intracellular granule distinct from histamine-containing secretory granules and lysosomes [12]. *Lxn* has about 30% sequence homology, but much greater structural homology, with tazarotene-induced gene 1 (*TIG1*) (or retinoic acid receptor responder 1, RARRES1), whose expression was down-regulated in an extensive list of tumor types in humans. [11], [13], [14], [15], [16]. *Lxn* and *TIG1* are closely linked genetically and may represent members of a family of functionally related genes. Moreover, *Lxn* was reported to be a TNF-responsive gene in human papillovirus-infected keratinocytes, suggesting that it may contribute to TNF-mediated suppression of cervical cancer development [17]. A recent report revealed that *Lxn* was down-regulated in patients with gastric carcinomas, and overexpression or knockdown of *Lxn* inhibited or stimulated tumor growth respectively [18]. Decreased or absent *Lxn* expression was observed in several human leukemia and lymphoma cell lines [19], as well as in malignant melanoma patients. [20] However, there is still lack of direct evidence for the cause-effect relationship between *Lxn* and hematopoietic malignancy.

Gene silencing, especially of tumor suppressors, and inappropriate gene activation, especially of oncogenes, are common themes in carcinogenesis. [21] Both often occur through aberrant DNA methylation that is accentuated during aging. [22], [23], [24], [25], [26], [27] A CpG-enriched region was identified in *Lxn* promoter and its hypermethylation was found in a variety of human gastric carcinoma cell lines. [18] Promoter hypermethylation of *TIG1* was also associated with its silencing in tumor cells. [13], [16] However, it is not known whether *Lxn* expression is regulated by promoter methylation in hematopoietic malignancy.

Molecular mechanisms underlying the role of *Lxn* in regulating hematopoiesis has not been determined yet. *Lxn* protein binds to carboxypeptidase A (Cpa) and inhibits its enzymatic activity, indicating that *Lxn* regulates protein degradation and metabolism. [19] *Lxn* was highly expressed in mast cells and its expression was further enhanced by LPS, indicating its potential function in inflammation. [11] Proteomic analysis on *Lxn* knock-out hematopoietic cells revealed that *Lxn* deletion reduced the abundance of multiple cellular proteins, especially those involving cell-stroma interaction, such as N-cadherin, Tie2, and Roundabout 4. [19] Ectopic expression of *Lxn* in gastric cancer cell lines led to the differential expressions of several cancer-related genes, including Maspin, WFDC1, SLPI, S100P, and PDGEFB [18], although none of them are overlapped with previously mentioned proteomic results. A recent study revealed ATP/GTP binding protein-like 2 (AGBL2) as a novel binding partner of TIG1. The interaction between TIG1 and AGBL2 regulates the microtubule tyrosination cycle, which is implicated in tumorigenesis, stem cell differentiation and development. [28] *Lxn* thus may have the similar mechanisms as TIG1 to regulate hematopoiesis.

Here, we provide evidence obtained from a variety of lymphoma and leukemia cell lines, as well as from primary cells from patients with these diseases, that *Lxn* expression is almost universally absent or significantly reduced from that of normal stem and progenitor cells. Moreover, treatment with a de-methylating agent at least partially restores *Lxn* expression in a variety of tumor cell lines, and methylation level of the CpG island in the *Lxn* promoter region is inversely associated with its expression. Perhaps most importantly, we show that when *Lxn* expression was re-initiated ectopically in two lymphoma cell lines using a retroviral expression vector, their growth, both *in vitro* and *in vivo*, was significantly blunted. We further show that *Lxn* inhibits lymphoma cell growth by significantly increasing apoptosis through the down-regulation of anti-apoptotic genes and that its anti-tumor activity is mediated via mechanisms unique from its canonical inhibition of carboxypeptidase A. These results demonstrate that *Lxn* plays a functional role in tumor cell growth and introduces an unexplored pathway potentially important to cancer treatment in patients.

## Results

### Loss of *Lxn* Expression in Malignant Cells

**Figure 1:**
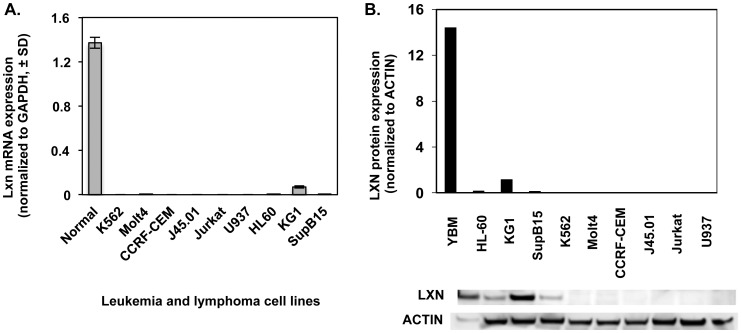
Decrease or loss of *Lxn* expression in leukemic and lymphoma cell lines. (**A**) *Lxn* mRNA expression in leukemia cell lines. *Lxn* mRNA level was measured by quantitative real-time PCR and shown as mean (±1 SD) (n = 12). Control is normal bone marrow (BM). (**B**) *Lxn* protein expression in leukemic cell lines measured by western blot. The blot (bottom) and their quantification (top) profiles demonstrate the absent or weak expression of LXN protein in leukemic and lymphoma cell lines. Actin was used as the internal control.

We first determined *Lxn* mRNA abundance in tumor cell lines by quantitative real-time PCR (Fig. 1A). Compared with normal primitive hematopoietic cells, *Lxn* mRNA expression was completely absent in a majority of tested leukemic lines, including K562, Molt4, CRF-CEM, J45.01, Jurkat and U937, and was significantly diminished in HL-60, KG-1 and SupB15 cell lines. LXN protein expression was also tested in all samples using Western blotting and nearly identical results were obtained in tested cell lines (Fig. 1B). Our results are consistent with those previously reported [19] and include additional leukemia and lymphoma cell lines.

### Aberrant Promoter Hypermethylation of *Lxn* in Hematopoietic Malignancy

**Figure 2:**
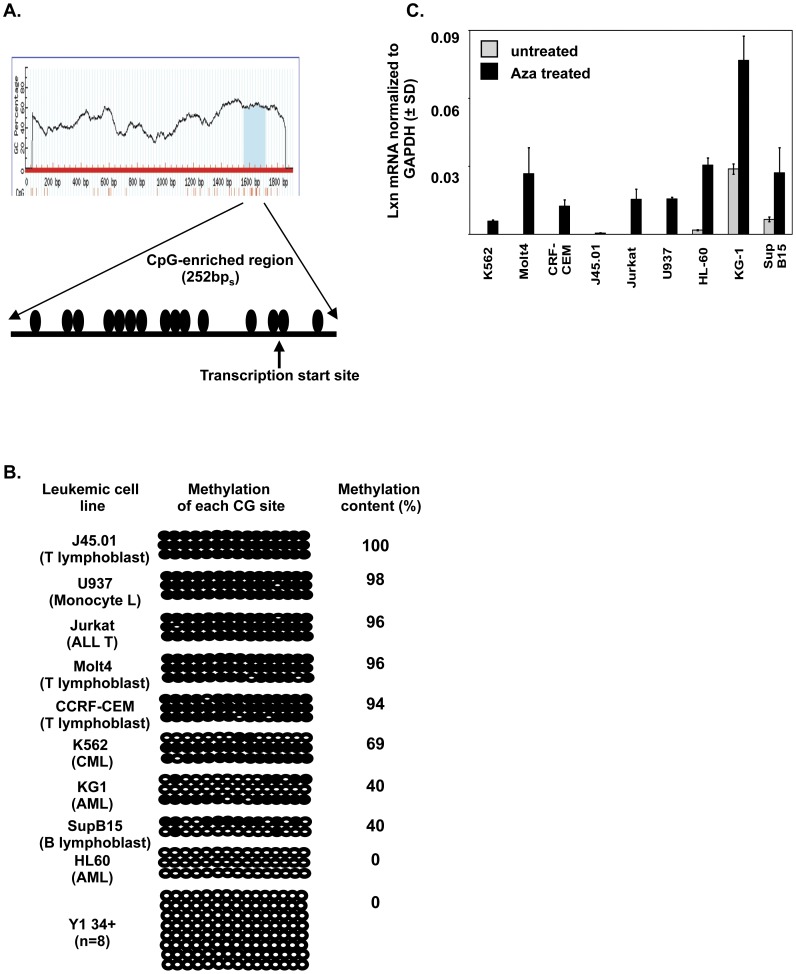
Hypermethylation of *Lxn* promoter CpG sites in leukemic cell lines. (**A**) CpG-enriched region in *Lxn* promoter. Sequence from 1500 base pairs upstream regulatory region to the first three exons was analyzed for CpG-enriched sites (top). A CpG island with 252 base pairs (bp_s_) length was identified. The transcription starting site is indicated. Each filled circle represents a CpG dinucleotide, and there are 15. (**B**) Bisulfite sequencing for *Lxn* promoter CpG island methylation analysis in leukemic cell lines. Files are each of the 15 CpGs and ranks are replicate clones sequenced for each CpG (n = 3). Open circles indicate unmethylated CpG (n = 3) dinucleotides. Filled circles indicate methylated CpG dinucleotides. The methylation content was quantified by dividing the number of methylated CpGs with total numbers of analyzed CpGs. (**C**) Restoration or up-regulation of *Lxn* expression by 5-aza-2′-deoxycytidine. *Lxn* mRNA was measured by quantitative real-time PCR in leukemic and lymphoma cell lines exposed with or without 2 uM 5-aza-2′-deoxycytidine for 4 days. Expression levels were normalized to endogenous control, *Gapdh*. Results shown are mean± SD of 12 replicates from 3 independent experiments.

To assess whether the loss or decrease of *Lxn* expression in malignant cell lines resulted from promoter hypermethylation, methylation of the 5′ CpG island of *Lxn* gene surrounding its transcriptional start site was determined by genomic bisulfite sequencing. The CpG island spans from within the canonical 5′ promoter (−208 nt) to the transcription start site (+1 nt), and extends through the entire first exon (+44 nt). There are 15 CpG dinucleotides within this region (Fig. 2A). The CpG island identified here is not exactly same as the one reported previously, this may be due to the different criteria we used for defining this region. Figure 2B depicts the quantitative variation in methylation for each CpG site among nine tumor cell lines. Almost complete methylation was seen in J45.01, U937, Jurkat, Molt4 and CCRF-CEM (>90%) lines, which commensurately showed an absence of *Lxn* expression. Scatteredly methylated CpG sites were found in K562, KG-1 and SupB15 lines, which were linked to the weak expression of *Lxn*. Surprisingly, although HL-60 had very low *Lxn* expression, we found that none of the CpG sites were methylated. This is probably due to other epigenetic mechanisms, such as histone deacetylation of latexin promoter in HL 60 cells.

### Reactivation of *Lxn* Expression with Demethylating Reagent Treatment

To test the hypothesis that *Lxn* promoter hypermethylation might be involved in the loss of expression in leukemia and lymphoma cell lines, we studied the effect of 5-aza-2′-deoxycytidine, a DNA demethylating reagent, on *Lxn* expression (Fig. 2C). After treatment with 2 uM 5-aza-2′-deoxycytidine for 4 days, *Lxn* gene expression was reactivated in cell lines completely lacking *Lxn* expression prior to treatment (K562, Molt4, CCRF-CEM, J45.01, Jurkat and U937), and was significantly up-regulated in HL-60, KG-1 and SupB15 lines. The up-regulation of *Lxn* by 5-aza-2′-deoxycytidine in HL60 cells, which shows no hypermethylation of CpG sties (Fig. 2B), might be because the demethylation of other DNA sequences induces chromatin remodeling, thus exposing *Lxn* promoter for the transcriptional initiation.

### Suppression of Growth of Mouse Lymphoma Cell Lines *in vitro* and *in vivo* Following Ectopic *Lxn* Expression

**Figure 3:**
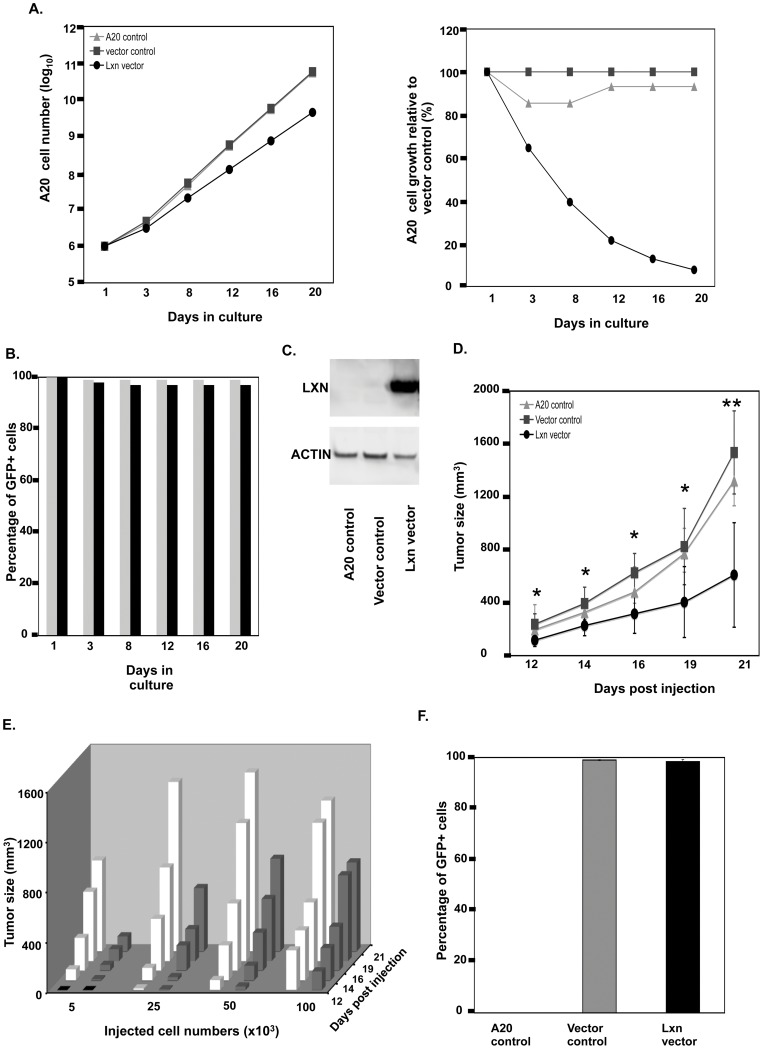
Overexpression *Lxn* suppresses growth of A20 lymphoma cell lines. (**A**) *In vitro* growth inhibitory effects of *Lxn* overexpression on A20 cells. A20 cells uninfected (A20 control) or infected with empty (vector control) or *Lxn*-containing vector (Lxn vector) were cultured for 20 days and counted by the trypan blue exclusion method. The absolute cell numbers at indicated time-points (X axis) were represented as log scale (Y axis) and were shown in left panel. The relative tumor growth in *Lxn*-overexpression A20 cells to controls was shown in the right panel. One representative experiment out of 3 independent ones is shown. (**B**) Proportion of GFP+ A20 cells infected with empty (light grey column) and *Lxn*-containing vector (black column) throughout 20 days of culture. Flow cytometric analysis was performed to detect GFP signal. (**C**) Western blot analysis with LXN antibody in total protein lysate from A20 cells at day 20 culture showing durable LXN overexpression. (**D**) *In vivo* tumor formation and growth with injection of 100,000 A20 cells. A20 cells were subcutaneously injected and tumor growth was monitored and measured by all three dimensions of tumors (Y axis) on days indicated on X axis. Shown is the compilation of 2 independent experiments with ± SD (n = 12). The statistical significance is represented by * (p<0.005) and ** (p<0.05). (**E**) *In vivo* tumor formation and growth with injection of graded doses of A20 cells. Graded doses of A20 cells (X axis) were subcutaneously injected and tumor growth was monitored and measured in all three tumor dimensions (Y axis) on days indicated on Z axis. Unfilled columns represent A20 cells infected with empty vector and filled columns represent cells with *Lxn*-containing vector. A representative experiment is shown (n = 4/group). (**F**) The fraction of GFP-expressing A20 cells in tumors biopsied at 21 days. Single tumor cell suspensions were made on day 21 post injection and subject to flow cytometric analysis for GFP-expressing cells.

To determine a correlative or causative relationship between *Lxn* expression and tumor development, we next asked whether or not the re-initiation of *Lxn* expression affected the growth rate of malignant cells *in vitro* and *in vivo*. To that end, we ectopically expressed *Lxn*, using a retroviral expression vector containing green fluorescent protein (GFP) marker, in the mouse BALB/c-derived A20 B lymphoma cell line, which lacks *Lxn* expression (Fig. 3C). The controls are A20 cells either un-infected or infected with empty vector. The GFP positive A20 cells were purified by flow cytometric cell sorting, and their growth was determined in vitro and in vivo.

Fig. 3 shows the potent effects of *Lxn* expression on inhibiting tumor growth *in vitro* and *in vivo*. Fig. 3A shows that cultures of A20 cells infected with the *Lxn* expression vector contained only about half the number of cells at day 3 as compared to A20 cells infected with the control (empty) vector or uninfected control cells (A20 control). The growth suppression by *Lxn* overexpression was exponentially amplified during subsequent days of culture and by day 20 nearly 16 fold less tumor cells were present in *Lxn*-overexpressing group than in control cultures (right panel). Fig. 3B shows that the fraction of GFP+ cells remained at 90–100% throughout the 20 days of culture in both the *Lxn* vector- or control vector-infected cells. Fig. 3C shows that at day 20 of culture neither uninfected A20 cells nor A20 cells infected with the control (empty) vector expressed detectable *Lxn* protein, whereas in the Western blot a strong *Lxn* band was evident in lysate of cells infected with the *Lxn* vector (upper band). LXN protein level at day 0 of culture in these cells is nearly identical to that at day 20 (data not shown). In a separate series of experiments, we have found a similar growth reduction *in vitro* when *Lxn* was ectopically expressed in WEHI231 lymphoma cells (data not shown).

Fig. 3D shows a compilation of two independent experiments in which ectopic *Lxn* expression suppressed the growth of A20 cell *in vivo*. 100,000 GFP+ A20 cells infected with either the *Lxn* expression vector or the GFP only control vector, or uninfected A20 cells, were injected subcutaneously into the flanks of BALB/c mice. Beginning at day 12 when tumors were first palpable, the *Lxn*-expressing cells caused significantly smaller tumors (filled circles; P<0.005). By day 21 when the experiments were terminated, the tumors in the *Lxn* vector-injected group averaged only 40% of the volume of tumors in the other two control groups (P<0.05, n = 12 in each group). At day 21, we determined the fraction of tumor cells expressing GFP and Fig. 3F shows that virtually all of the tumor cells in the *Lxn* and control vector groups were GFP+. More to the point, Western blots confirmed strong expression of *Lxn* as in the cells analyzed after 20 days in culture (Fig. 3C). Thus, the reduction in tumor growth was due to durable *Lxn* expression in the tumor cells themselves.

To explore the effects of cell dose on tumor size and latency before being palpable, we ejected graded doses of *Lxn*-expressing or control A20 cells (Fig. 3E) to BALB/c recipient (n = 4/group). At the 5,000 and 25,000 inoculum sizes, the *Lxn*-expressing cells not only resulted in more impressive suppression of the size of tumors than with the 100,000 cell inoculum in Fig. 3D, but resulted in delayed onset of measurable-sized tumors. At day 21, the reduction in tumor size caused by ectopic *Lxn* expression was 83% and 63% at the 5,000 and 25,000 cell doses, respectively. Host animals were necropsied for evidence of gross metastases to spleen, thymus and liver. No evident tumors were found in any of the treatment groups. Similarly, flow cytometry detected no GFP+ cells in these anatomical sites (data not shown).

### *Lxn* Inhibits Tumor Cell Growth through Increasing Apoptosis but not via its Canonical Function

**Figure 4:**
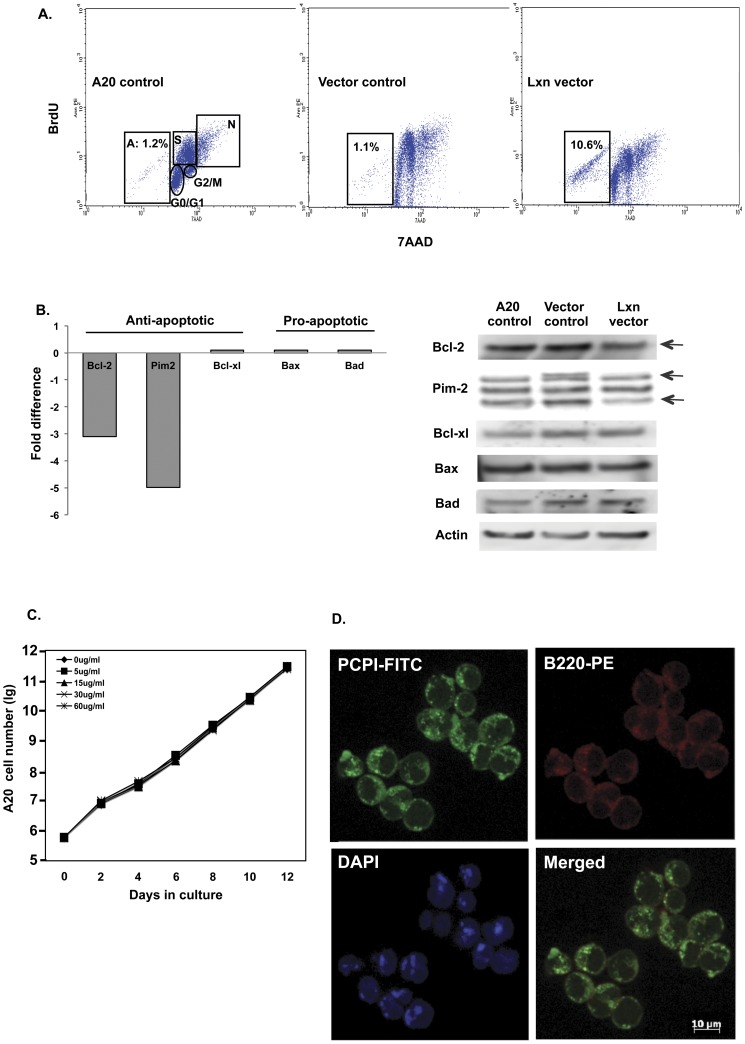
Ectopic *Lxn* expression increases apoptosis of A20 cells. (**A**) Flow cytometric analysis of cell cycle and apoptosis in cultured A20 cells. A20 cells without (A20 control) or with empty (vector control) or *Lxn*-containing vector (Lxn vector) were cultured for 21 days. BrdU and 7AAD staining and flow cytometric analysis was used for detecting different phases of the cell cycle (G0/G1, S, G2/M) and apoptotic (A) and necrotic (N) population. A representative flow cytometric file from Day 5 of culture is shown. (**B**): Down-regulation of anti-apoptotic genes by *Lxn* overexpression. Apoptosis PCR arrays were performed on A20 cells infected with empty or *Lxn*-expressing vectors. Left panel shows the fold changes in the mRNA expression of several selected genes (as indicated in X axis) in *Lxn*-overexpressing cells compared to the control. Western blot confirms the decreased expression of Bcl-2 and two isoforms of Pim-2 at the protein level in *Lxn*-overexpressing cells (arrowhead). The other apoptosis-related genes, such as Bcl-xl, Bax and Bad, did not show significant difference in mRNA and protein expression. (**C**) The growth curve of A20 cells treated with graded doses of potato carboxypeptidase inhibitor (PCPI). No significant difference is detected between the control and any of the doses of PCPI. (**D**) Internalization of PCPI to cytosol of A20 cells. A20 cells were cultured with fluorescein isothiocyanate (FITC) labeled PCPI. Shown is a three-color micrograph with PCPI in green (FITC), the B220 lymphoid cell surface marker in red (phycoerythrin, PE), the nucleus in blue (DAPI), and merged image of red and green as indicated.

As we previously reported in our normal hematopoietic stem cell studies, high *Lxn* expression is associated with increased apoptosis and decreased proliferation. To determine if these mechanisms were applied to our results with tumor cells, we next measured these two parameters in vector-infected A20 cells throughout 21 days of culture. As shown in Fig. 4A, flow cytometric analysis of cells stained with BrdU and 7AAD allows for the discrimination of cell subsets that are apoptotic (A), necrotic (N) or reside in G0/G1, S and G2/M phase of cell cycle. By day 5 of culture, 10 fold more *Lxn*-expressing tumor cells were undergoing apoptosis than control cells (10.6% vs 1%). NO significant differences in the proliferation and the numbers of necrotic cells were observed between *Lxn*-overexpressing and control cells (data not shown). These results point to apoptosis as the major cellular mechanism in *Lxn*-mediated tumor suppression. We next investigated the molecular mechanisms underlying *Lxn*-induced apoptosis. We performed apoptosis pathway-specific PCR arrays in A20 cells infected with either empty or *Lxn*-expressing vectors. We found that 17 out of 84 apoptosis-related genes were differentially expressed in *Lxn*-overexpressing cells, and majority of them (15 genes) were down-regulated (data not shown). We specifically examined several well-known apoptotic genes, such as Bcl-2, Pim-2, Bcl-xl, Bax, and Bad, and plotted their expression in Fig. 4B. Two anti-apoptotic genes, Bcl-2 and Pim-2, were down-regulated by at least 3-fold in *Lxn*-overexpressing cells (left panel) as compared to the control, whereas pro-apoptotic genes, including Bax and Bad, did not show any change in their mRNA levels. Western blots of these proteins observed the similar reduction in Bcl-2 and Pim-2 (all three isoforms) but not in Bcl-xl, Bax and Bad in *Lxn*-overexpressing A20 cells (right panel), which is consistent with PCR array result. Thus our data indicate that *Lxn*-induced apoptosis in A20 cells is mainly through the down-regulation of anti-apoptotic regulators, such as Bcl-2 and Pim-2.

A well-known function of *Lxn* is its role as the sole carboxypeptidase A (CPA) inhibitor in mammalian cells. There are six *Cpa* genes (1–6) in this family. In order to test whether or not the suppressive effect of ectopic *Lxn* expression on the growth of A20 and WEHI231 cells was due to its canonical inhibitory activity, we carried out the following experiments. We first determined which *Cpa* genes are enriched in hematopoietic stem/progenitor cells. By searching our gene expression profile of hematopoietic stem/progenitor cells (unpublished data), we found that *Cpa3* is the only gene highly expressed in hematopoietic cells (Table S1). Next, we tested *Cpa3* expression in A20 cells that were uninfected, or infected with empty or *Lxn* expression vectors, and found that it was not expressed in any type of these cells (Figure S1). Last, we treated A20 and WEHI cells with potato carboxypeptidase A inhibitor (PCPI), a 39 amino acid protein which strongly inhibits mammalian CPA, at a series of concentrations ranging from 5 to 60 ug/ml of culture medium. As seen in Fig. 4C, none of the concentrations had any effect on the growth patterns of the tumor lines, despite the continuous presence of PCPI for 12 days of culture. To rule out the possibility that PCPI failed to inhibit tumor growth because it did not enter the cells, we used fluorescein isothyiocyanate (FITC) to label PCPI in cells. Fig. 4D shows A20 cells following a 30 min incubation with labeled PCPI (30 ug/ml of culture medium). The three-color micrograph shows PCPI in green (FITC), the nucleus in blue (DAPI), and the B220 lymphoid cell surface marker in red (phycoerythrin, PE). It is apparent that PCPI is plentiful in the cytosol but is not found in the nucleus. The doses of PCPI chosen for the above experiments were taken directly from a study in which the inhibitor was shown to inhibit the growth of pancreatic adenocarcinoma cells by directly interfering with the epidermal growth factor signaling pathway [29]. Maximal growth inhibition was achieved at 30–50 ug/ml. Thus, the mechanisms by which *Lxn* regulates both stem cell population size and lymphoma growth inhibition reside in a novel pathway involving apoptosis.

### Down-regulation of *Lxn* in Primary Leukemia and Lymphoma Cells

**Figure 5:**
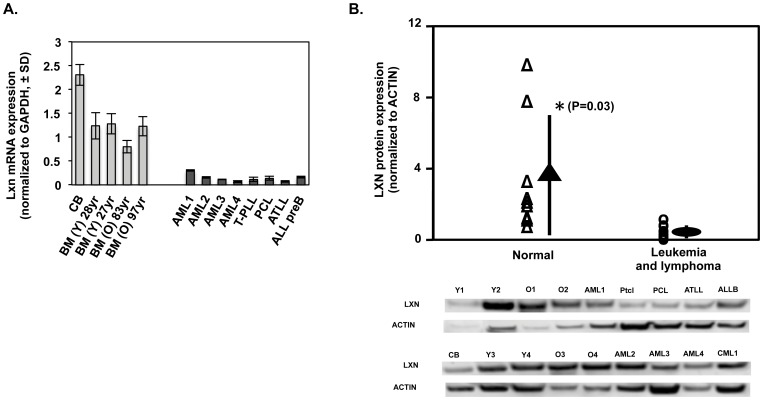
Decreased *Lxn* expression in stem/progenitor-enriched CD34+ cells in patients with leukemia and lymphoma. (**A**) *Lxn* mRNA expression in bone marrow and peripheral blood CD34+ cells from leukemia and lymphoma patients and normal individuals. *Lxn* mRNA level was measured by quantitative real-time PCR and shown as mean (±1 SD) (n = 12). Normal samples were derived from cord blood (CB), bone marrow (BM) of young at 31 (Y1) and 39 (Y2) years old and old at 85 (O1) and 97 (O2) years old people. The patient samples include acute myeloid leukemia (AML), T cell pro-lympho leukemia (Pctl), plasma cell leukemia (PCL), acute T cell lymphoma (ATLL) and acute lymphoid leukemia (ALL, preB phenotype). (**B**) LXN protein expression in bone marrow and peripheral blood CD34+ cells from leukemia and lymphoma patients and normal individuals. Western blot was performed on the corresponding samples shown in panel (**A**) plus five more samples, including two young (Y3 and Y4; at 40 and 31 years old respectively), two old (O3 and O4; at 80 and 87 years old respectively) and chronic myeloid leukemia (CML). The blots (bottom) and their quantification (top) profiles demonstrate the significantly decreased Lxn protein level in leukemic CD34+ and lymphoma cells (P = 0.03).

Based on the strong evidence for the tumor suppressive function of *Lxn* in leukemia and lymphoma cell lines, we asked whether or not *Lxn* expression is altered in primary cells derived from patients with malignancies. We isolated stem/progenitor-enriched CD34+ cells from bone marrow and blood cells in lymphoma and leukemia patients, as well as in normal donors. The patients included those with acute myeloid leukemia (AML), T cell pro-lymphocytic leukemia (T-PLL), plasma cell leukemia (PCL), acute T cell lymphoma (ATLL) and acute lymphoid leukemia (ALL, preB phenotype). The normal CD34+ cell samples were derived from cord blood (CB) and young (31 and 35 years) and old (85 and 97 years) adults. *Lxn* mRNA expression, quantified by real-time PCR, was decreased by at least two-thirds in primary malignant CD34+ cells (Fig. 5A). Quantification of LXN in CD34+ cells of all human normal and leukemic samples compiled to date is plotted in Fig. 5B. The result shows a significant decrease of LXN protein expression in malignant cells (P = 0.03) even though *Lxn* expression at the protein level is more variable than that at the mRNA level. Two additional old samples in the normal control group contribute to this variation, which is consistent with the report that the number and functionality of HSCs in old individuals, perhaps stem cell regulatory gene(s), showed more dramatic variations as compared to their young counterparts [30]. In summary, these data indicate that *Lxn* dysregulation may be involved in human hematological malignancies. In the future study, it will be interesting to examine the expression of two anti-apoptotic genes, Bcl-2 and Pim-2, in patient samples to see whether or not they are up-regulated.

## Discussion

Recently we found that *Lxn* is a natural regulator of hematopoietic stem cell population in mice by influencing self-renewal and apoptosis [4]. *Lxn* expression in stem cells is inversely proportional to the size of the population in mice and thus acts as a negative regulator through cell-intrinsic mechanisms. The observation that low *Lxn* expression in hematopoietic cells was associated with increased replication led us to hypothesize that *Lxn* was down-regulated in malignancies with high proliferative rates and/or low apoptosis.

Here, we show that *Lxn* is either not expressed or is strongly down-regulated in a variety of leukemias and lymphomas. As a result of these findings and close linkage structurally and genetically with TIG1, we proposed that *Lxn* may similarly act as a tumor suppressor. Along with this proposal, there is expectation that manipulation of *Lxn* levels in malignant cells would alter their growth characteristics. Accordingly, two lymphoma cell lines in which *Lxn* expression was absent were infected with a *Lxn* expression vector. In one of the most significant findings of this study, we found that re-initiation of *Lxn* expression dramatically reduced their growth *in vitro* and *in vivo*. These results demonstrate that the level of *Lxn* expression is functionally related to both normal and malignant cell growth and, if the findings are true for human tumors, may provide an avenue for therapeutic intervention. Surprisingly, *Lxn* knock-out mice did not development leukemia nor lymphoma even though their hematopoietic stem cell pool was over-expanded and blast cells were detected in the peripheral blood [19]. These results suggest that *Lxn* may influence the crucial, step in carcinogenesis [31]. It thus will be of great interest to introduce additional carcinogenetic “hits”, such as oncogenes or alkylating agents, to *Lxn* knockout stem cells to determine whether *Lxn* deletion will accelerate tumor development. In addition, we observed that re-initiation of *Lxn* expression did not completely suppress tumor cell growth, suggesting that it would at best be a part of a multi-drug regimen.

We investigated the possible mechanisms by which ectopic *Lxn* expression may suppress growth of lymphoma cells and found increased apoptosis to be the major mechanism causing inhibition. Consistent with our study of normal hematopoietic cells, our recent findings indicate that high *Lxn* content is associated with more apoptotic cells, thus fewer stem cells [4]. Apoptosis is controlled by the balance of both positive (pro-apoptotic) and negative (anti-apoptotic) signals. We found that *Lxn*-induced apoptosis is mainly through the down-regulation of anti-apoptotic genes, such as Bcl-2 and Pim-2. Decreased level of Bcl-2 allows other pro-apoptotic proteins such as BAX and BAK to aggregate, inducing cytochrome C release and caspase activation, and apoptosis. Dysregulation of Bcl-2 is implicated in a variety of hematopoietic malignancies. [32] Three Pim-2 protein isoforms are active kinases which phosphorylate pro-apoptotic protein BAD and dissociate its binding from Bcl-xL, resulting in the inhibition of Bad-induced cell death. [33], [34] Enhanced expression of Pim-2 was detected in multiple B-cell lymphoma types [35], T-cell lymphoblastic leukemia/lymphoma [36], and acute myeloid leukemia [37]. Thus our studies identify *Lxn* as a novel regulator of the apoptotic pathway, in which it suppresses tumor survival through the down-regulation of Bcl-2 and Pim-2 anti-apoptotic factors.

The well- known function of *Lxn* is the sole carboxypeptidase A inhibitor in mammals. [12] Therefore, it was plausible that other carboxypeptidase A inhibitors might mimic *Lxn*’s anti-tumor effects. A CPA inhibitor from potato tubers has been shown to be an effective inhibitor of mammalian CPA and was therefore chosen to test its anti-tumor properties. We found that it freely entered A20 lymphoma cells but did not have suppressive growth effects via apoptosis, irrespective of dose. Since CPAs are not expressed in A20 cells, we conclude that non-canonical functions of *Lxn* were responsible, yet unknown intracellular functions of *Lxn* account for its anti-tumor effects. In addition, it is unlikely that tumor suppressive effect of *Lxn* is through the regulation of ABGL2-mediated microtubule tyrosination cycle, like TIG1, because we did not detect the expression of ABGL2 in hematopoietic cells (data not shown). These results are in line with findings of other investigations in which gene expression or protein abundance regulated by *Lxn* were not related to carboxypeptidase inhibition [18], [19]. Thus, the action of mode of *Lxn* in regulating normal and malignant hematopoiesis is not through its canonical CPA inhibitor activity. Future studies aimed at identifying direct targets, or downstream signaling pathways, of *Lxn* will be critical for our understanding of its function in normal and malignant hematopoiesis.

Addressing the issue of why and how *Lxn* expression is down-regulated in tumor cells, we found that CpG dinucleotides in regulatory regions of the *Lxn* gene were methylated. Most human leukemia and lymphoma cell lines tested showed strong patterns of methylation involving most of the 15 CpG dinucleotides in *Lxn* promoter. This, in turn, led to a loss of gene expression that was at least partially reversible. When cell lines were treated with de-methylating reagent, 5-aza-2′-deoxycytidine, *Lxn* expression was re-initiated or up-regulated. Thus, hypermethylation of the CpG island in the *Lxn* promoter may contribute to silencing or down-regulation of *Lxn* expression in leukemia and lymphoma cells. Ongoing studies will focus on the genetic and epigenetic regulation of *Lxn* transcription in cancer stem cells, with promise for improved targeted approaches for cancer prevention, diagnosis, and therapy.

## Materials and Methods

### Animals

Young 8-to 12- week old female C57BL/6 (B6) and 7-week old female BALB/c mice were purchased from the Jackson Laboratories (Bar Harbor, ME). Mice were kept in the animal facilities of the University of Kentucky under pathogen-free conditions according to NIH-mandated guidelines for animal welfare. They were fed with acidified water and food *ad libitum*. All animal work in this study was approved by Institutional Animal Care and Use committee (IACUC) at the University of Kentucky (Identification number: 2010-0753).

### Leukemia Cell Lines

Nine human leukemic cell lines (K562, Molt4, CCRF-CEM, J45.01, Jurkat, U937, HL-60, KG-1 and SupB15) and two mouse lymphoma cell lines (WEHI-231 and A20) were included in our study. Human leukemic cell lines were purchased from American Type Culture Collection (ATCC) (Manassas, VA). Mouse lymphoma cell lines are gifts from Dr. Bondada [38]. The leukemia cell lines were maintained in IMDM supplemented with either 10% (K562) or 20% (KG-1, HL-60 and Sup-B15) fetal bovine serum (FBS), or RPMI medium with 10% FBS, 10 mM Hepes (Molt4, CCRF-CEM, J45.01, Jurkat and U937), 0.05 mM 2-mecaptoethanol, 80 U/mL penicillin, and 80 mg/mL streptomycin. The cells were incubated in a humidified atmosphere of 5% CO2 in air at 37°C.

### Isolation of CD34+ Cells

Primary AML cells were obtained from the peripheral blood and bone marrow of patients at the Markey Cancer Center and Northwestern University. Normal bone marrow was obtained as discarded material following pathologic analysis, surgical marrow harvest, or from the National Disease Research Interchange (NDRI). CB was obtained from patients at the University of Kentucky Obstetrics Department or from the NDRI. All tissues were obtained with the approval of the respective institutional review boards and appropriate informed consent (confirmation number is 11-0315-F3R from University of Kentucky). Some samples were collected form Northwestern University and transferred to University of Illinois at Urbana-Champaign with approval of Material Transfer Agreement (MTA). We processed 9 normal and 9 leukemia and lymphoma samples, the age and disease type of each sample was described in the figure 5 legends. Marrow and blood cells were depleted of erythrocytes by suspending in 150 mM NH4Cl plus 10 mM NaHCO3 for 5 minutes, followed by 2 washes with phosphate-buffered saline (PBS). Blood cells were subjected to Ficoll-Paque (Pharmacia Biotech, Piscataway, NJ) density gradient separation to isolate the mononuclear white blood cell compartment. Resulting leukocytes from marrow or blood were then used for immunoaffinity selection and flow cytometric sorting. For CD34+ cell selection, the Miltenyi immunoaffinity device (VarioMACS) was used according to the manufacturer’s instructions (Miltenyi Biotech, Auburn, CA), and further purified by immune-staining with anti-CD34 antibodies (Pharmingen, San Diego, CA) and sorting in a triple-laser FACSVantage flow cytometer (Becton Dickinson Immunocytometry Systems, San Jose, CA). In some cases, leukocytes were cryopreserved at a concentration of 5×10^7^ cells/mL in freezing medium consisting of Iscoves modified Dulbecco medium (IMDM), 40% fetal bovine serum (FBS), and 10% dimethyl sulfoxide (DMSO).

### Quantitative Real-time PCR

To measure the expression of *Lxn* in leukemic cells, quantitative real-time PCR was performed. Identical numbers (200,000) of cells were used for total RNA extraction using RNeasy Mini kit (QIAGEN, Valencia, CA) according to the manufacture’s instruction. Isolated total RNA was reverse transcribed into cDNA using random hexamers in a TaqMan® reverse transcription solution (PN N8080234) and stored at −20°C. In real-time PCR reactions, primer and probe mix for LXN (human and mouse) were purchased from Applied Biosystems (Foster city, CA, USA). TaqMan® human glyceraldehyde-3-phosphate dehydrogenase (GAPDH) was served as an endogenous control to normalize LXN expression. PCR reactions were set up according to manufacturer’s instructions using TaqMan® universal PCR master mix (PN 4304437). Analyses of gene expression were performed in single reporter assays in an ABI PRISM 7700 sequence detection system (PE Biosystems, Foster city, CA, USA).

RT^2^ Profiler Apoptosis PCR Array (PAMM-012Z, QIAGEN) was performed according to the manufacture’s instructions. cDNAs was prepared from A20 cells infected with empty or lxn expression vectors with RT^2^ First Strand cDNA Kit (C-03, QIAGEN) and added to RT^2^ qPCR master mix (PA-012). The mixture was aliquoted across the PCR array which contains 84 apoptosis-related genes. Gene expression and quantification was performed in an ABI PRISM 7700 sequence detection system, and data was analyzed with using web-based software (http://pcrdataanalysis.sabiosciences.com/pcr/arrayanalysis.php). The genes with more than 2-fold change in expression and showing statistically significant (p<0.05) were chosen for candidate gene.

### Western Blots

Cell samples were lysed at a concentration of 2×10^7^ cells/ml in a protein lysis buffer containing: 10 mM Tris pH 7.5, 50 mM NaCl, 30 mM sodium pyrophosphate, 50 mM NaF, 5 µM ZnCl2 and 1% Triton X-100, 2.8 ug/ml aprotinin (Sigma-Aldrich; St. Louis, MO), 1 mM phenylmethylsulfonyl fluoride (Sigma), 1 mM sodium vanadate (Na3VO4) 1 ug/ml pepstatin, and 1 µg/ml leupeptin (Oncogene Research, MA, USA). Lysate was incubated on ice for 30 min, and then centrifuged at 15,000×g for 10 minutes to remove debris. The resulting supernatant was then aliquoted and stored at −80°C. For Western blot, protein lysates were thawed and mixed with running buffer and a reducing agent (Novex, San Diego, CA, USA, per manufacturer’s instructions) and heated at 95°C for 5 minutes. Samples were then analyzed by denaturing PAGE (Novex, 10% bis-Tris gel) using the equivalent of 4×10^5^ cells per lane. Following electrophoresis, samples were electro-transferred onto immunobilon-P membranes (Millipore, Bedford, MA, USA), which were subsequently blocked and probed with polyclonal rabbit anti-LXN Ig-G antibody at a 1∶3000 dilution. This antibody was generated from the *Lxn*-specific amino acid sequence CKHNSRLPKEGQAE at the carboxyl terminus, and was produced by Bethyl Laboratories, Inc. (Montgomery, TX). The antibody for detection of human LXN was purchased from Abcam Inc. (Cambridge, MA) and used at 1∶2000 dilution. The antibodies for apoptotic proteins, Bcl-2, Bcl-xl, Bax, Bad were provide by Dr. Bondada. The antibody for Pim-2 was purchased from eBioscience (San Diego, CA). Primary antibodies were detected using alkaline phosphatase-conjugated secondary antibodies (Santa Cruz Biotechnology) and electro-chemifluorescent (ECF) reagent (Pharmacia Biotech) according to the manufacturer’s instructions. Blots were visualized using a Molecular Dynamics STORM 860 system and Imagequant Software. Following the detection and quantification of anti-LXN antibody, immunobilon-P membrane was sequentially stripped in 40% methanol and the buffer containing 100 mm ß-mercaptoethanol, 2% sodium dodecyl sulfate and 62.4 mM Tris-HCl to remove ECF reaction product and antibodies, respectively. The stripped membrane was re-probed with anti-actin antibody (Sigma) at 1∶500,000 dilution and detected as described previously.

### Genomic Bisulfite Sequencing

To investigate the methylation pattern of *Lxn* promoter, CpG island analysis in the upstream sequence of *Lxn* open reading frame. Nucleotide sequence of *Lxn* in upstream region (–1000 bp) and the first 3 exons (+373 bp) was obtained from Ensembl database (www.ensembl.org) with ID number ENSG00000079257. CpG island search using CpG island searcher website (http://www.uscnorris.com/cpgislands2/cpg.aspx) showed a 252 bp region (–208 bp to +44 bp) in upstream of *Lxn* sequence enriched for CpG repeats. The criteria of 5 CpG island is: GC content >50%, ratio of CpG to GpC >0.6 and 200 bp of minimum length. Genomic DNAs were isolated using AquaPure Genomic DNA kit (Bio-Rad, Hercules, CA) and modified by sodium bisulfite using EpiTect® Bisulfite kit (QIAGEN, Valencia, CA). For the *Lxn* promoter methylation study, we designed primers that could amplify a 423 bp fragment in the upstream region of *Lxn* containing CpG island. The forward primer sequence is 5′ GTTGGTGTTTGATAAGTATGTGG 3′, and the reverse primer sequence is 5′ TTTAACCTTCTACACCTCAAACAC 3′. The annealing temperatures for primers were 52°C for 2 minutes. Hot-start PCR with a total cycle number of 30 was used in all PCR amplifications. Denaturation and extension cycles were maintained for 95°C, 30 seconds and 72°C, 1 minutes respectively. The amplified fragments were cloned into the pCR2.1-TOPO vector using TOPO TA Cloning Kit (Invitrogen Carlsbad, CA) and sequenced (MWG Technology) (n≥ 3 clones for cell line and n≥ 8 clones for primary cells).

### 5-aza-2′-deoxycytidine Treatment

To examine the correlation of promoter hypermethylation and *Lxn* gene expression, the leukemia cell line, shown to have a lack or decrease of *Lxn* expression, was subject to 5-aza-2′-deoxycytidine treatment. Cells were plated with 2 uM 5-aza-2′-deoxycytidine (Sigma-Aldrich; St. Louis, MO) and incubated for 4 days. The medium and the drug were replaced every 24 hours (hrs.) and cells were harvested for RNA and DNA extraction 4 days after treatment.

### Infection of WEHI231 and A20 Cells with *Lxn* Expression Vector

Cloning of the mouse *Lxn* gene into Sfbeta 91 retroviral vector and production of viral supernatant were performed exactly as described previously [4]. WEHI-231 and A20 cells were infected by 10 ml viral supernatant at a density of 1×10^6^ cells per 10 cm plate along with 4 µg/ml of polybrene for 48 hours. The infected cells (GFP+ cells) were sorted and expanded in culture medium. The expanded GFP+ population, if not used immediately, was cryopreserved at a concentration of 1×10^7^ cells/mL in freezing medium consisting of 80% fetal bovine serum, and 20% dimethyl sulfoxide (DMSO).

### Measurement of Growth of Retrovirally-transduced Tumor Cells

Sorted GFP+ A20 cells over expressing *Lxn* or Sfbeta 91 empty vector were counted on a hemacytometer using trypan blue dye exclusion and 500,000 cells were seeded into 25 cm^2^ tissue culture flask in 4 mls media. Cells were incubated in a humidified atmosphere of 5% CO2 in air at 37°C and subsequently counted on days 3, 8, 12, 16 and 20. At each time point, cells were split and maintained at a concentration of 500,000 cells per 4 ml media. The cumulative cell number was calculated from the cell counts and the dilutions made at each culture split. FACS analysis was also performed at each time-point to measure the percentage of GFP+ cells. For the in vivo measurement of tumor cell growth, various numbers (5,000; 25,000; 50,000 and 100,000) of GFP+ A20 cells over expressing *Lxn* or Sfbeta 91 empty vector were injected in a 50 µl bolus subcutaneously in the shaved flank of BALB/cJ mice given 3.0 Gy of gamma radiation 4 hrs. prior. Lymphomas were detectable by palpation 10–12 days post-injection and all three dimensions of tumors were measured blind with calipers on days 12, 14, 16, 19, and 21. The same individual made the measurements from day-to-day without knowing the treatment regimen the mice received. At day 21, host mice were euthanized, the lymphomas were excised, and single cell suspensions were made of each to determine the fraction of tumor cells expressing GFP.

### Cell Cycle and Apoptotic Analysis

The culture of GFP+ A20 cells was maintained as described above. At each time-point, cell cycle analysis was measured by BrdU labeling using BrdU Flow Kit (Pharmingen, San Diego, CA) according to the manufacturer’s instruction. 10 ul of BrdU solution (1 mM) was added to 1×10^6^ cells in 1 ml culture medium and incubated for 1 hour. The cells were fixed and permeabilized by Cytofix/Cytoperm Buffer and treated with 30 ug DNase for 1 hour at 37°C. After washing with Perm/Wash buffer, cells were stained with PE-conjugated anti-BrdU antibody for 20 minutes at room temperature, washed and 20 ul of 7-AAD was added. The cells were analyzed by flow cytometry on Facscan (Becton Dickinson Immunocytometry Systems, San Jose, CA).

### Immunohistochemstry of A20 Cells Treated with Potato Carboxypeptidase Inhibitor (PCPI)

PCI was purchased from Sigma-Aldrich Co. (St. Louis, MO). FITC labeling of PCI was performed by using FLUOROTAG™ FITC CONJUGATION KIT (Sigma-Aldrich) according to manufacturer’s instruction, and was used to treat A20 cells for fluorescence internalization assays. Cells are cultured on 22×22 mm microscope cover glasses (Fisher Scientific Co., Pittsburgh, PA) in media as described above. Cells were fixed onto cover glasses with 1∶1 methanol:acetic acid and washed 3 times by PBS. Cells were incubated with FITC-conjugated PCI at a concentration of 30 ug/ml at 37°C for 30 minutes, washed and stained with phycoerythrin (PE)-conjugated B220 and DAPI. The cover glass coated with A20 cell monolayer was flipped immediately and sealed onto glass slide. The image was taken with a Zeiss Axiovert- 200 microscope using a high-resolution Zeiss digital camera (Carl Zeiss Inc., Thornwood, NY).

### Culture of A20 Cells with Potato Carboxypeptidase Inhibitor

To determine the effects of PCPI on A20 cell growth, A20 cells were seeded at a density of 6×10^5^/well in 6-well plates, cultured overnight before the addition of 0, 5, 15, 30 or 60 ug/ml PCI. The cells were fed every 2 days with fresh medium (as above) containing the respective concentration of PCI, and viable cells were counted on a hemacytometer using trypan blue dye exclusion. Cells were split according to cell population size to maintain a cell concentration of 2–5×10^6^/ml and cultures were maintained for 12 days. The cumulative cell number was calculated from the cell counts and the dilutions made at each culture split.

### Statistical Analysis

Data were analyzed by either student *t*-test assuming unequal variance with *P*<0.05 (two-tail), or a one-way ANOVA.

## Supporting Information

Figure S1 ***Cpa3*** **is not expressed in A20 cells.** Real-time PCR was performed on A20 cells that were either uninfected (A20 control) or infected with empty (vector control) or *Lxn* expression vector (Lxn vector) to quantify *Cpa3* mRNA expression. The amplification plots for *Lxn* (**A**), *Cpa3* (**B**) and *Gapdh* (**C**) transcript show that *Lxn* is highly expressed in A20 cells infected with *Lxn* expression vector whereas A20 and vector controls have very low expression levels, consistent with the results in Fig. 3c. *Cpa3* transcript is not amplified in all types of cells, indicating it is not expressed in A20 cells. These figures show the amplification plots of four individual biological replicates (n = 4) for each gene.
(TIF)

Table S1 **Carboxypeptidase A3 (Cpa3) is highly expressed in stem/progenitor cells.** Expression level of CPAs were measured by microarray on a bone marrow population null for cell markers characteristic of lineage-specific differentiated blood cells (Lin-negative), and positive for the Sca-1 and c-Kit cell markers (LSK) cells, enriched for hematopoietic stem/progenitor cells in mouse. The isolation of LSK cells as well as microarray analysis was performed as previously described [38] except the array platform is mouse Genome 430 2.0 Array (Affymetrix). Three biological samples were assayed, and the mean expression value (mean_y_B6) for each gene in the table is the average of three readings. Cpa3 (highlighted in gray) is the only *Cpa* gene that is highly enriched in LSK cells. *Gapdh* is the endogenous control.
(PDF)
