# Supplementary figures and images for: Latexin Is Down-Regulated in Hematopoietic Malignancies and Restoration of Expression Inhibits Lymphoma Growth
Source: PLoS One. 2012 Sep 27;7(9):e44979. doi: 10.1371/journal.pone.0044979 (PMC3459965; doi:10.1371/journal.pone.0044979)

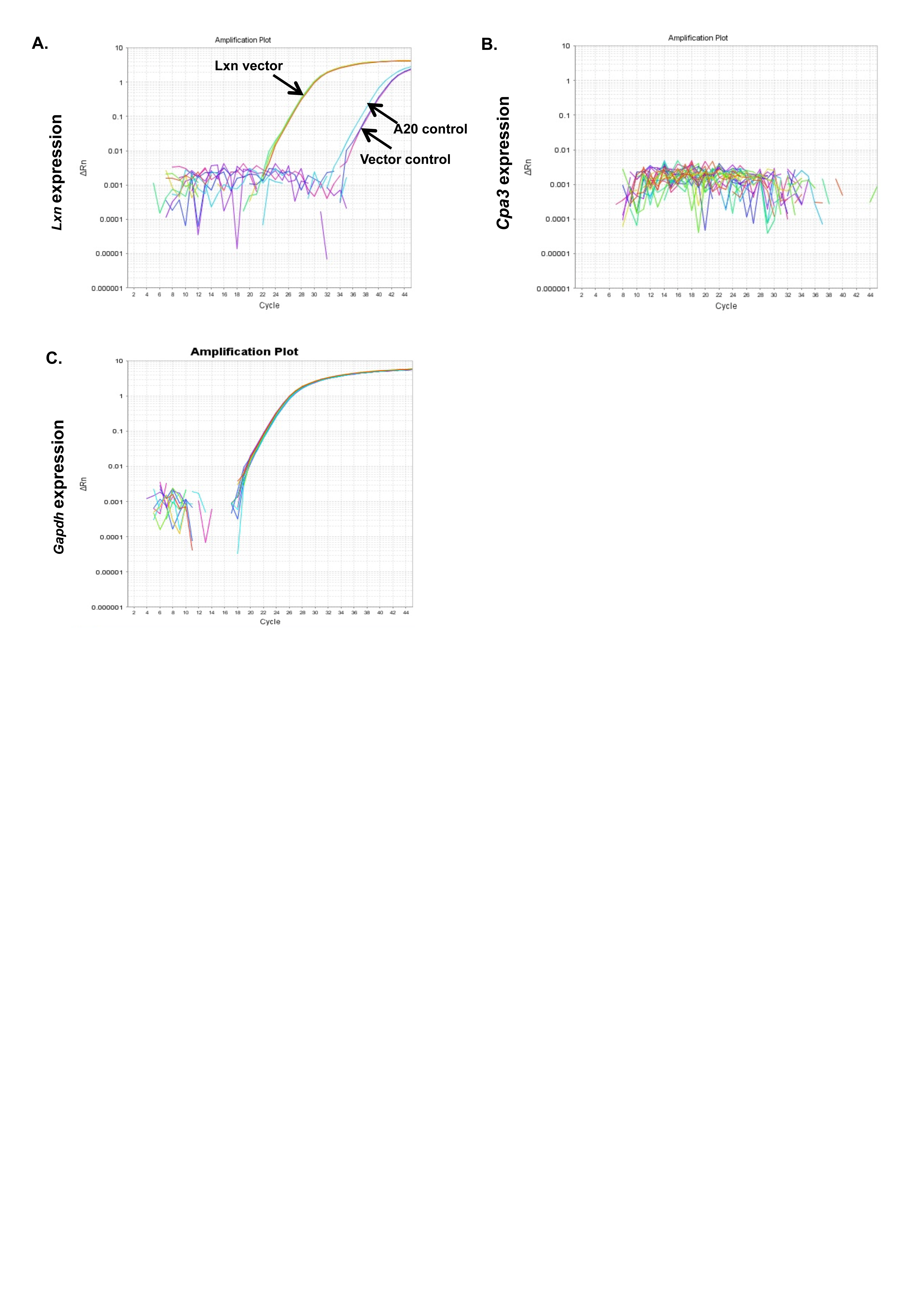

Supplement: Figure S1 — Cpa3 is not expressed in A20 cells. Real-time PCR was performed on A20 cells that were either uninfected (A20 control) or infected with empty (vector control) or Lxn expression vector (Lxn vector) to quantify Cpa3 mRNA expression. The amplification plots for Lxn (A), Cpa3 (B) and Gapdh (C) transcript show that Lxn is highly expressed in A20 cells infected with Lxn expression vector whereas A20 and vector controls have very low expression levels, consistent with the results in Fig. 3c. Cpa3 transcript is not amplified in all types of cells, indicating it is not expressed in A20 cells. These figures show the amplification plots of four individual biological replicates (n = 4) for each gene. (TIF) [file pone.0044979.s001.tif]
