# Supplementary material for: Latexin Is Down-Regulated in Hematopoietic Malignancies and Restoration of Expression Inhibits Lymphoma Growth
Source: PLoS One. 2012 Sep 27;7(9):e44979. doi: 10.1371/journal.pone.0044979 (PMC3459965; doi:10.1371/journal.pone.0044979)
Supplement: Table S1 — Carboxypeptidase A3 (Cpa3) is highly expressed in stem/progenitor cells. Expression level of CPAs were measured by microarray on a bone marrow population null for cell markers characteristic of lineage-specific differentiated blood cells (Lin-negative), and positive for the Sca-1 and c-Kit cell markers (LSK) cells, enriched for hematopoietic stem/progenitor cells in mouse. The isolation of LSK cells as well as microarray analysis was performed as previously described [38] except the array platform is mouse Genome 430 2.0 Array (Affymetrix). Three biological samples were assayed, and the mean expression value (mean_y_B6) for each gene in the table is the average of three readings. Cpa3 (highlighted in gray) is the only Cpa gene that is highly enriched in LSK cells. Gapdh is the endogenous control. (PDF) [file pone.0044979.s002.pdf]

Probeset	1418625_s_at	1448730_at	1453770_at	1427995_at	1440617_at
Gene Title	glyceraldehyde-3-phosphate dehydrogenase	carboxy-peptidase A3, mast cell	carboxy-peptidase A4	carboxy-peptidase A5	carboxy-peptidase A6
Gene Symbol	GAPDH	Cpa3	Cpa4	Cpa5	Cpa6
Chromosomal Location	6 C 59.3cM	3 A2|3 13.2 cM	6 A3.3	6 A3.3	1 A2
Entrez Gene	14433	12873	71791	74649	329093
mean_y_B6	11982.87	391.33	20.43	16.17	11.43
GO Biological Process Description	glucose metabolism /// glycolysis	proteolysis and peptidolysis	proteolysis and peptidolysis	proteolysis and peptidolysis	proteolysis and peptidolysis
GO Cellular Component Description	cytoplasm /// mitochondrion	extracellular space	extracellular space	---	---
